# Kompensation und Stabilisierung: Corona, der Staat und die Soziale Marktwirtschaft in Deutschland

**DOI:** 10.1007/s41025-021-00215-6

**Published:** 2021-03-17

**Authors:** Paul Nolte

**Affiliations:** Friedrich-Meinecke-Institut, Freie Universität Berlin, Berlin, Deutschland

**Keywords:** Ordnungspolitik, Neo-Keynesianismus, Austerität, Neoliberalismus, Wohlfahrtsstaat, Economic Governance, Neo-Keynesianism, Austerity, Neo-liberalism, Welfare state

## Abstract

Soziale Marktwirtschaft bezeichnet für die Geschichte der Bundesrepublik keine reale, sondern eine diskursive Ordnung. In der Corona-Pandemie folgte die Politik bislang einer hyperkeynesianischen Prämisse: Der Staat sieht sich in der Verantwortung, die ökonomische Krise bis zur Unspürbarkeit auszugleichen. Die Kosten dieser Stabilisierungspolitik sind noch nicht erkennbar. Die Corona-Krise kann zum Katalysator eines Ausbaus des Sozialstaats und der Umwelt- und Klimapolitik werden.

## Abstract (en)

Social Market Economy does not mean a real order, but an order of discourse. So far, during the Corona crisis German politics followed a hyper-Keynesian assumption: The state feels responsible to balance economic effects so widely that they are no longer perceptible. The costs of this politics of stabilisation are incalculable. The Corona crisis will accelerate the expansion of the welfare state and foster ecological and climate politics.

Die Corona-Pandemie hat im Jahre 2020 eine globale Erschütterung ausgelöst, die sich auf nahezu alle Bereiche des gesellschaftlichen Lebens erstreckt. Einschränkungen der persönlichen Bewegungsfreiheit und der privaten Kontakte gehören ebenso dazu wie ein Herunterfahren des Kulturlebens, Grenzen für die freie Religionsausübung und Beschränkungen der wirtschaftlichen Tätigkeit, die daraus teils unmittelbar folgen: Wenn nicht mehr gereist wird, steht das Geschäft mit der Mobilität, ob für Reisebüros, Hotels oder Fluggesellschaften, vor dem Kollaps. Ist es sinnvoll, in dieser globalen und totalen Krise nach dem Einfluss von Corona auf die Soziale Marktwirtschaft in Deutschland zu fragen?

Gewiss nicht deshalb, weil die Auswirkungen der Pandemie an nationalen Grenzen Halt machten. Schon eher lohnt die Frage, ob in den politischen, auch wirtschaftspolitischen „Maßnahmen zur Eindämmung des Corona-Virus“ nationale Muster und Besonderheiten erkennbar sind, die sich aus früheren Erfahrungen, aus spezifischen institutionellen Mustern oder der besonderen politischen Kultur eines Landes speisen. In Deutschland war diese Krise auch die Stunde der Länder und des Föderalismus, aber insgesamt markierte sie zunächst einmal die Stunde des Nationalstaats, als Grenzen geschlossen wurden und das Schengen-Regime vorübergehend außer Kraft trat. Das änderte sich im Sommer mit der Macron-Merkel-Initiative für ein Hilfspaket der Europäischen Union, auch mit der koordinierten Impfstrategie am Ende des Jahres. Doch noch immer stehen, auch innerhalb der EU, die nationalen Gesellschaften und ihre politischen Führungen im Zentrum des Krisenmanagements, selbst wenn sich die Werkzeuge der verschiedenen Staaten oft ähneln.

Ebenso wenig signalisiert die Frage nach dem Nexus von Pandemie und Sozialer Marktwirtschaft die Präferenz für eine unternehmerische oder kapitalistische Perspektive: Wenn nur die Wirtschaft, wenn nur der Profit keinen Schaden nimmt! Vielmehr waren einige der frühen und elementaren Krisenerfahrungen geradezu Lehrstücke über das Funktionieren der Wirtschaft für spätere Schulbücher. Als der Gastwirt, die Friseurmeisterin oder der Cellist, die zugleich meine Nachbarn sind, kein Geschäft mehr machten, entstand eine kaum für mögliche gehaltene Empathie mit dem dichten, angesichts der öffentlichen und medialen Konzentration auf Großunternehmen sonst meist unsichtbaren und unterschätzten Gewebe des Marktes. Der Begriff der Lieferkette erfuhr eine ungeahnte Konjunktur – weil plötzlich klar wurde, dass das Ineinandergreifen von Zahnrädern der Zulieferung, Produktion, Logistik und Distribution nicht kalte Profitmaschine, sondern Schmiermittel des Alltagslebens ist. Auch die Vakzine wurden in marktwirtschaftlichem Wettbewerb erforscht und hergestellt und nicht nach staatlichem Plan in einem Zentrallabor. Die Väter der Sozialen Marktwirtschaft hätten allen Grund zur Freude: An vielen Stellen regiert der Wettbewerb – wichtiger noch, die Ordnung der Wirtschaft wurde in der Krise nicht als ein separates (und womöglich feindliches) kapitalistisches Subsystem begriffen, sondern wurde als Ordnung der Gesellschaft, ihres Alltags, ihrer unmittelbaren, auch nicht-ökonomischen bzw. nicht-marktlichen Lebensvoraussetzungen weithin erfahrbar.

Auch in dieser Diagnose jedoch geht das Thema „Corona und die Soziale Marktwirtschaft“ noch nicht auf. Im Zentrum steht vielmehr dies: Ökonomische Folgen und politische Reaktionen in der Pandemie haben Grundregeln der Marktwirtschaft in Frage gestellt, nicht zuletzt durch einen massiven Schub der Staatsintervention und der staatlichen Substitution für Märkte – sei es für Arbeit, Dienstleistungen oder anderes – zur Bewältigung oder Abfederung der Krise. Wer schon in den letzten Jahren Zweifel daran hegte, ob das Ideal oder die Spielregeln der Sozialen Marktwirtschaft im Sinne Walter Euckens, Alfred Müller-Armacks oder Ludwig Erhards in der Bundesrepublik noch gelten, wird im gegenwärtigen keynesianischen und staatsinterventionistischen Schub endgültig die Totenglocke dieser Ordnung läuten hören. Wenn es je einen diskursiven Konsens über die Soziale Marktwirtschaft gab – am Anfang des 21. Jahrhunderts jedenfalls nicht mehr. Für eine erste historische Vermessung des Corona-Impacts auf die politischen Ökonomien westlicher Gesellschaften muss man sich zuerst Klarheit verschaffen, auf welche wirtschaftliche Ordnung das Virus gestoßen ist. Erst dann lässt sich sinnvoll nach Kontinuitäten und Strukturbrüchen, nach unmittelbaren Wirkungen und Langzeitfolgen fragen.

## Die Soziale Marktwirtschaft vor der Corona-Krise

Die Bemühungen von politischen Parteien, Stiftungen und Think Tanks reißen nicht ab, die Soziale Marktwirtschaft als ein reales Fundament der Bundesrepublik Deutschland zu verstehen und von dort nach ihrer Transformation, ihrer Zukunftsfähigkeit, ihrer zeitgemäßen Anpassung zu fragen. In historischer und kulturwissenschaftlicher Perspektive hat sich jedoch längst gezeigt, dass „Soziale Marktwirtschaft“ weniger eine reale als eine diskursive Ordnung bezeichnet. Der Begriff ist zur normativ aufgeladenen Chiffre geworden, zur Projektionsfläche ganz unterschiedlicher wirtschaftlicher, politischer und gesellschaftlicher Zielvorstellungen, als positives Ideal ebenso wie als Negativfolie. Wer, aus eher linker Perspektive, die globale Wirtschaft seit den 1980er-Jahren kritisch als Wende zu „Neoliberalismus“ und „Marktradikalismus“ versteht, wird in Deutschland mehr Erfolg auf dem Meinungsmarkt haben, wenn er an die Soziale Marktwirtschaft erinnert, statt ein sozialistisches Wirtschaftssystem zu fordern. Wer umgekehrt die Auffassung vertritt, das wesentliche Kennzeichen der Gegenwart sei eine staatsinterventionistische und wohlfahrtsstaatlich-sozialdemokratische Politik, der sich auch die Partei Ludwig Erhards zunehmend ausgeliefert habe, meint mit demselben Appell an die Soziale Marktwirtschaft etwas ganz Anderes: die Rückkehr zu mehr Markt und Wettbewerb und die Beschränkung des Staates bzw. des Regierungshandelns auf die formale Regelsetzung für die Akteure am Markt.

Solche Deutungskonflikte sind in offenen Gesellschaften üblich, doch im Falle der Sozialen Marktwirtschaft ist das Spektrum breiter, der Konsensraum schmaler als bei anderen Grundbegriffen. Über die Bedeutung und Prinzipien von „parlamentarischer Demokratie“ etwa wird man schneller Einigkeit erzielen können. Wie eben skizziert, kann man eine eher „ordoliberale“ und eine eher „sozialstaatliche“ Interpretation der Sozialen Marktwirtschaft unterscheiden – so, dass man geradezu von den zwei Sozialen Marktwirtschaften sprechen könnte. Darin kommen nicht nur gegenwärtige gesellschaftspolitische Differenzen zum Ausdruck. Die beiden Sichtweisen haben vielmehr Wurzeln in der deutschen Geschichte seit dem Kaiserreich, in der Begründung des Konzepts in der Mitte des 20. Jahrhunderts ebenso wie in der Transformation polit-ökonomischer Ordnung (nicht nur) der Bundesrepublik seitdem (Plumpe und Scholtyseck 2012; Abelshauser 1996).

Die Ordoliberalen haben Recht, dass Soziale Marktwirtschaft nicht eine um den Sozialstaat – im Sinne dessen, was die internationale Forschung „welfare state“ nennt – ergänzte Marktordnung meinte. Das Adjektiv-Attribut zielte vielmehr, einerseits, auf die breite gesellschaftspolitische, ja ethische und normative Einbettung der wirtschaftlichen Ordnung. Historisch war das, schon in der Historischen Schule der deutschen Nationalökonomie, (auch) eine Antwort auf den Marxismus. Andererseits meinte „sozial“ zugleich die staatliche Regulierung, und zwar in einem doppelten Sinne: nicht als sozialstaatliche Transferleistungen, sondern als ordnungspolitische Regelsetzung für den Wettbewerb, aber auch – und das vergessen die „Ordoliberalen“ heute bisweilen – als Selbstverständlichkeit staatlicher Eingriffe und Begrenzungen, ja staatlicher Lenkung in Bereichen der wirtschaftlichen Aktivität, die in diesem Denken geradezu als nichtökonomische (nämlich im weitesten Sinne ethische) Voraussetzung des Wirtschaftens ausgeklammert blieben (Abelshauser 2004, S. 93–100). Daher bedurfte es des „Neoliberalismus“, um die Privatisierung bzw. Vermarktlichung von Infrastrukturleistungen wie Post und Telekommunikation, Schienen- und Luftverkehr durchzusetzen. Langwierige Kämpfe bis ins frühe 21. Jahrhundert waren nötig, bis in Deutschland Ladenschlusszeiten fielen oder Busunternehmen mit den Fernzügen der Bahn in Wettbewerb treten konnten.

Diese im Sinne des 19. Jahrhunderts geradezu „staatswirtschaftlichen“ Muster waren mithin kein Produkt des Keynesianismus, der in den 1960er und (frühen) 1970er-Jahren in der Bundesrepublik seinen Höhepunkt erlebte, sondern Teil einer ordnungspolitischen Vision, die auf ältere deutsche Traditionen zurückgriff, von der Historischen Schule der Nationalökonomie bis zu dem großen Schub der Ent-Privatisierung der Wirtschaft nach dem Ersten Weltkrieg. Seit den späten 1960er-Jahren – vielleicht kann man sagen: seit der ersten Großen Koalition 1966–69 und dem Bündnis von Karl Schiller und Franz Josef Strauß – setzte sich zunehmend ein anderes Verständnis von Sozialer Marktwirtschaft durch, das von dem späteren Paradigmawechsel vom Keynesianismus zum Neoliberalismus bemerkenswert unberührt blieb: nämlich die schon erwähnte „Markt-cum-Wohlfahrtsstaat“-Variante. Gerade in Deutschland stand, auch seit den 1980er-Jahren und in der Kanzlerschaft Helmut Kohls und anders als in den angelsächsischen Ländern, das eine nicht gegen das andere. Statt eines „dismantling the welfare state“ (Pierson 2010) erlebte die Bundesrepublik gewiss ein Abflachen der sozialstaatlichen Expansion, aber auch ein Umsteuern sozialstaatlicher Prioritäten, etwa auf die Familienförderung oder in die Pflegeversicherung. In der Wiedervereinigung expandierte der Sozialstaat ohnehin gewaltig, vor allem durch die Übertragung der Sozialversicherungssysteme auf die neuen Länder.

Die Expansion marktlicher, wettbewerblicher und privatwirtschaftlicher Elemente seit den 1980er-Jahren ist völlig unstrittig, aber sie ging in Deutschland grosso modo nicht auf Kosten des Sozialstaates, sondern mit dessen weiterer Expansion einher. Dieses Muster setzte sich, nach dem Intermezzo der „Agendareformen“ zu Beginn der 2000er Jahre, in den Großen Koalitionen der letzten Legislaturperioden fort, zuletzt etwa mit der Einführung des Mindestlohns 2015 und der Grundrente 2020. Oft übersehen oder unterschätzt wird, in welchem Umfang die Europäische Union in dieser Phase zum „neoliberalen“ Treiber wettbewerblicher Prinzipien geworden ist, nicht nur in der (De‑) Regulierung von Waren‑, Dienstleistungs- und Finanzmärkten, sondern auch, zum Beispiel, in der Wissenschafts- und Forschungspolitik (Patel 2018). In Deutschland bildete sich in den ersten beiden Jahrzehnten des 21. Jahrhunderts eine Variante wirtschaftlicher Ordnung bzw. eine Politische Ökonomie heraus, die man sehr wohl als Neoliberalismus kennzeichnen kann, die von den marktliberalen Varianten Großbritanniens und der USA seit den 1980er-Jahren jedoch weit entfernt ist (Nolte 2019). Ihr Kennzeichen ist ein hohes Maß an staatlicher Regulierung, nicht selten geradezu: von staatlicher Induzierung des Wettbewerbs. Dabei reicht die Institutionalisierung wettbewerblicher, pseudo-marktlicher Verfahren weit über die „Wirtschaft“ im engeren Sinne hinaus und verbindet sich umstandslos mit Staatsexpansion. Die deutsche „Exzellenzinitiative“ für die Universitäten drückt dieses spezifische Syndrom besonders prägnant aus: Sie ist ein Amalgam aus Marktlichkeit und vermehrter Staatsintervention, ja expansiver Staatsfinanzierung als „Ersatz-Profit“ für die Akteure des Wettbewerbs, die ja nicht wirklich am Markt agieren, also etwa preislich um Studierende konkurrieren dürfen.

Ist dieses Janusgesicht noch ein Wiedergänger der Sozialen Marktwirtschaft aus dem Gründungsjahrzehnt der Bundesrepublik? Präziser gefragt: ist sie ein Resultat deutscher Pfadabhängigkeit in der politischen Ökonomie? Man kann das durchaus so sehen. Komplizierter wird die Beschreibung, nimmt man die fiskalische Dimension hinzu. Auf den ersten Blick entspricht die antikeynesianische Austeritätsposition, die Deutschland in den letzten zwei Jahrzehnten besonders prononciert vertreten hat, der Interpretation linker Kritiker des Neoliberalismus: staatliche Zurückhaltung statt „deficit spending“, Angebots- statt Nachfrageorientierung. Bei näherem Hinsehen irritiert jedoch nicht nur, dass ausgerechnet die USA sich auch unter republikanischen Präsidenten keine Zurückhaltung in der Expansion des Bundeshaushalts durch Schuldenaufnahme auferlegt haben. Vor allem konnte die „schwarze Null“ ihre (nicht nur politische, sondern auch kulturelle und normative) Durchschlagskraft in Deutschland nur als eine „schwarz-grüne Null“ erlangen, nämlich unter dem Vorzeichen fiskalischer „Nachhaltigkeit“ im Blick auf die kommenden Generationen. Dieser ökologische Antikeynesianismus, dessen komplexe Triebkräfte von seinen amerikanischen Kritikern wie Paul Krugman kaum wahrgenommen werden (Krugman 2012), speist sich zu einem guten Teil aus ethischen Impulsen, die in der Kontinuität der frühen Sozialen Marktwirtschaft stehen. Umso größer ist die Herausforderung dieses Modells, wenn es in der Corona-Krise durch einen sprunghaften, scheinbar ohne Obergrenzen hantierenden Ausbau der Staatsverschuldung zur Disposition gestellt wird. Gewiss geschieht das im breiten und parteiübergreifenden Konsens. Aber die „Bazooka“ von Olaf Scholz ist, zeitgeschichtlich gesehen, auch der Returnschlag zum Bündnis von Wolfgang Schäuble und Kerstin Andreae.

## Politische Ökonomie und Soziale Marktwirtschaft in der Corona-Krise

Seit dem Frühjahr 2020, seit dem ersten „Lockdown“ in Deutschland (und ungefähr zeitgleich vielen anderen Staaten Europas) Mitte März, hat sich das Verhältnis zwischen Staat und Wirtschaft grundlegend verändert. Der Staat – vor allem der Bund, in geringerem Ausmaß auch Länder und Kommunen – sind in eine neue Form von Verantwortung getreten, die sich anders als in früheren Krisen bzw. Krisen anderen Typs nicht bloß aus einer allgemeinen moralisch-sozialpolitischen Verpflichtung ergibt. In einem Sozialstaat gilt, dass den Schwächsten geholfen werden muss, unabhängig davon, woher diese Schwäche rührt. Die Ursachen mögen, wie in der klassischen agrarischen Ökonomie, im wörtlichen Sinne naturbedingt sein (Wetter, Missernten, Armut und Hunger); sie mögen in strukturellen, z. B. technologisch getriebenen Veränderungen der Wirtschaft liegen (Produktivitätswachstum, Deindustrialisierung, Freisetzung von Arbeitskräften); oder auch in unternehmerischen Fehlentscheidungen (Managementfehler oder falsches Marktkalkül, Insolvenz, Arbeitslosigkeit). In der Sozialen Marktwirtschaft deutscher Prägung würde man vor allem so argumentieren: Menschen sind als Wirtschaftssubjekte frei in ihren Präferenzen, und das gilt nicht zuletzt für ihre Rolle als Konsumenten. Wenn sich kollektive Präferenzen ändern, zum Beispiel Fernreisen mit dem Flugzeug wegen ökologischer Bedenken vermieden werden, können Unternehmen wie Luftfahrtgesellschaften in die Krise geraten. Dann greifen in der Sozialen Marktwirtschaft drei unterschiedliche institutionelle Hebel.

Erstens ist es den Tarifpartnern vorbehalten, Abfederungsregeln auszuhandeln, etwa einen betrieblichen Sozialplan. Während das originär kein Handlungsbereich des Staates war, ist er an dieser Stelle zunehmend direkt beteiligt worden; Stichworte sind Transfergesellschaften, Transferkurzarbeitergeld und Insolvenzgeld. Zweitens war es in der Sozialen Marktwirtschaft schon immer staatliche Aufgabe, in der Umstrukturierung von Märkten (zum Beispiel beim Ausfall von „Big Players“ oder bei Gefahr der Konzentration von Marktmacht) ordnungspolitisch auf faire Wettbewerbsbedingungen im krisenhaften Übergang zu achten. Ferner übernimmt es der Sozialstaat, drittens, individuelle Lebensschicksale in solchen Fällen abzufedern (Arbeitslosenversicherung, Grundsicherung u. a.). Einen Grenzfall stellte, historisch gesehen seit vielen Jahrhunderten und bis in die „Hartz IV“-Debatten unserer Tage, die sogenannte „selbstverschuldete Armut“ dar: In welchem Umfang ist der Staat (also die Solidargemeinschaft der Steuerzahler) zur Hilfe verpflichtet, wenn die selbstständige Bestreitung des Lebensunterhalts möglich ist, aber von den Betroffenen nicht geleistet wird?

Es ist wichtig, sich diese historisch gewachsenen und von Pfadabhängigkeiten (wie der deutschen Sozialen Marktwirtschaft oder des europäischen Sozialmodells) geprägten Grundkonstellationen klarzumachen, um die Besonderheit der Corona-Situation seit dem Frühjahr 2020 zu verstehen. Denn in historisch durchaus singulärer Weise kompensiert in ihr der Staat nicht primär die Fehlleistungen (oder, wertneutraler gesagt: Verhaltensänderungen) Dritter (des Wetters, von Managern, von Konsumenten), sondern ist selber der unmittelbare Verursacher der wirtschaftlichen Disruptionen. Das wirft in ganz anderer Weise die Frage der Kompensationsverpflichtung auf. Gewiss leidet die Wirtschaft unter „dem Coronavirus“, unter „der Pandemie“ – das ist der mittelbare Verursacher, eine naturgegebene „Plage“ wie die Heuschrecken des Alten Testaments oder die Hungersnot in Europa nach dem Ausbruch des Vulkans Tambora 1816/17 (Behringer 2015). Auch das gab und gibt es in der Corona-Pandemie: Unternehmen, die aus eigener Erwägung ihre Geschäftstätigkeit reduzieren, um sich an kurzfristig veränderte Marktbedingungen anzupassen. Dominierend und neuartig ist jedoch ein anderes Muster: nämlich staatliches Handeln und politische Beschlüsse, die unmittelbar das Handeln von Akteuren am Markt einschränken – am schärfsten im Lockdown-Regime, also in der mandatierten Schließung von Ladengeschäften, Dienstleistungsbetrieben und Kultureinrichtungen. Daraus erwächst nicht nur eine neue Form der moralischen Verantwortung für die Folgen (mit Appellen, der Staat „müsse etwas tun“), sondern in einem Rechtsstaat auch eine justiziable Verantwortung, die möglicherweise eingeklagt werden kann. In der Regel eilt der Staat möglichen Regressforderungen aber, wie sich im letzten Jahr gezeigt hat, durch seine Zusagen bereits voraus.

Diese neuartige „Kompensationsschuld“ in der Folge staatlicher Maßnahmen zur Bekämpfung der Pandemie ist eine von zwei großen Determinanten, von zwei Säulen der neuartigen Corona-Ökonomie des Jahres 2020. Die andere ist die Prämisse der systemischen Stabilisierung. Wir haben es zwar mit einer ungeheuerlichen Krise zu tun, mit einem Jahrhundertereignis. Die Folgen sollen jedoch weitestgehend abgefedert, wenn nicht bis zur Unsichtbarkeit ausgeglichen werden. Die unausgesprochene Prämisse ist die Wahrung und Fortschreibung des status quo ante, der wirtschaftlichen und gesellschaftlichen Strukturen des Jahres 2019. Unter der Annahme einer kurzfristigen Disruption, wie noch im Frühjahr 2019, war das unmittelbar einleuchtend: Warum sollte man, wie es in Ländern mit stärker marktkapitalistischen Arbeitsmarktstrukturen wie den USA tatsächlich geschah, Millionen Menschen in die Arbeitslosigkeit gehen lassen, wenn man sie bereits sechs oder acht Wochen später an genau derselben Stelle wieder braucht? Also machte Deutschland, wie viele europäische Nachbarn, von erheblich erweiterten Kurzarbeitsregelungen Gebrauch. Dabei spielte, wie auch in anderen Aspekten, die Erfahrung der Krisenbearbeitung aus der Finanzkrise von 2008/09 eine zentrale Rolle. Warum sollte eine Fluggesellschaft in die Insolvenz gehen, wenn sie einige Monate später wieder ihren vollen Flugplan anbieten könnte? Also wurde die Bundesrepublik Deutschland Teilhaber der Lufthansa und diese damit ein teilstaatliches Unternehmen. Also reduziert die Deutsche Bahn ihren Fahrplan nicht so, wie sie es unter Marktbedingungen tun müsste, weil sie im Kern immer ein staatliches Unternehmen geblieben ist.

Das oberste staatliche Gebot, aber eben auch der gesellschaftliche und kulturelle Konsens in dieser Krise scheint es zu sein, das Krisenhafte, insbesondere soweit es ökonomische Folgen zeitigt, möglichst unsichtbar werden zu lassen. Nicht für jede einzelne Ladeninhaberin, nicht für jeden Musiker, aber für 83 Mio. Menschen insgesamt soll „business as usual“ gelten, wenn schon soziale Kontakte und außerökonomische Dimensionen der Lebensführung wie die Begegnung mit Familie und Freunden eingeschränkt sind. Genau darin liegt ein Kern des staatlichen Versprechens in der Coronakrise: Du magst stark eingeschränkt sein, aber ärmer wirst Du nicht! Man kann darüber spekulieren, welche historischen Erfahrungen, welche Muster kollektiver Erinnerung diesem Versprechen des Staates ebenso wie solcher Erwartung in der Bevölkerung zugrunde liegen. In Deutschland stößt man dann auf die Traumata der Zwischenkriegszeit, auf die Inflation nach dem Ersten Weltkrieg und auf die Weltwirtschaftskrise der frühen 1930er-Jahre mit ihren politisch destabilisierenden Wirkungen. Man denkt zudem an die Notsituation nach dem Zweiten Weltkrieg und ihre Überwindung im „Wirtschaftswunder“. Das mag der deutschen Stabilitätssehnsucht eine besondere Prägung geben, und ihrer bisweilen trivial-keynesianischen Grundstimmung, deren warnendes historisches Exempel immer noch der damalige Reichskanzler Heinrich Brüning ist, wenn es heißt, gegen die Krise könne man „nicht ansparen“. Auch gehörte das Bemühen um einen gesellschaftlichen Ausgleich, um die Moderierung von Konflikt und Ungleichheit, selber zu den Grundprinzipien der Sozialen Marktwirtschaft als ordnungs- und gesellschaftspolitisches Programm (Nolte 2000). Am Beginn des 21. Jahrhunderts, in einer globalen Pandemie und angesichts ähnlicher Reaktionsweisen selbst in den USA mit ihren „stimulus checks“, erst recht in Europa, liegt die tiefere Ursache aber wohl eher in den über viele Wohlstandsjahrzehnte gewachsenen Erwartungen an demokratische Regierungen, dass auch in der schlimmsten Krise der Lebensstandard nicht merklich eingeschränkt werden dürfe.

Man könnte von einer hyperkeynesianischen Prämisse der Krisenbewältigung sprechen: Krisen kann man nicht nur (mit Keynes) staatlich und wirtschaftspolitisch bekämpfen, etwa durch „deficit spending“. Der Staat ist vielmehr in der Verantwortung, ökonomische Krisen bis zur Unspürbarkeit auszugleichen. Mehr noch, es wird bisweilen erwartet, dass er die Bürger für die schmerzhaften außerökonomischen Einschränkungen finanziell entschädigt, etwa durch die Senkung der Mehrwertsteuer, die vor allem dem Geschäft der Verkäufer zugunsten kommen sollte, in einer Konsum- und Konsumentengesellschaft aber unweigerlich zum Recht der Verbraucher auf niedrigere Steuern umgedeutet wird. Kompensation und Stabilisierung, diese beiden Prämissen ergänzen sich und finden ihren besonders markanten Ausdruck in der staatlichen Bereitschaft, vom Lockdown betroffenen Gewerbetreibenden bis zu 75 % des Vorjahresumsatzes zu erstatten. Wie auch immer zeitlich befristet und mehrfach konditioniert diese Regelung ist, so ist sie doch ebenso ordnungspolitisch außergewöhnlich wie historisch einzigartig. Sie verführt geradezu zu dem Gedankenexperiment einer staatsorganisierten Para-Marktwirtschaft, in der die Unternehmen zwar überwiegend in privater Hand sind, ihren Erfolg am Markt aber nicht mehr in der Konkurrenz beweisen müssen, sondern in Umsatzäquivalenten staatlich garantiert und ausgezahlt erhalten.

Dabei ergeben sich zwei Fragen: erstens nach der Finanzierung dieser und ähnlicher „Bazooka“-Maßnahmen; zweitens nach dem Verhältnis von Konservierung und Strukturwandel in der Krise. Was die Finanzierung angeht, hat sich die Politik, wiederum im Einklang mit gesellschaftlichen Erwartungen, frühzeitig darauf festgelegt, dass ohnehin von der Krise betroffene Menschen diese nicht auch noch im Portmonee spüren dürften. Marktwirtschaftliche oder fiskalische Alternativen sind kaum diskursfähig. Da die Schließung von Läden und die Einschränkungen der Reisefreiheit und des Tourismus das Geldvermögen der privaten Haushalte rasch und erheblich haben anwachsen lassen, läge die Abschöpfung durch höhere Steuern nahe: Dann würde die Kompensation des Friseursalons nicht aus Krediten bezahlt, die in späteren Jahren oder von späteren Generationen getilgt werden müssen, sondern wäre eine Solidarabgabe derjenigen, die den regelmäßigen Haarschnitt, oder die dritte und vierte Urlaubsreise des Jahres, einmal ausfallen lassen müssen.

Während diese Option im linken Spektrum durchaus erwogen wird (mit der charakteristischen Einschränkung auf die „Reichen“, nicht auf die Gesamtheit derer, die Konsumausgaben sparen), ist keine politische oder gesellschaftliche Kraft erkennbar, die sich zur alternativen Finanzierung der expansiven Corona-Maßnahmen für eine vorübergehende Senkung von Löhnen und Gehältern stark machen würde, sei es im Öffentlichen Dienst, sei es im weiteren tariflichen Konsens auch in der Privatwirtschaft. Dahinter winkt nicht nur erneut das Brüning-Trauma, sondern auch eine spezifische Corona-Stilisierung, mit der den Deutschen die Krise psychisch erträglicher gemacht werden soll: Ob Pflegekraft, Lehrerin oder Paketzusteller – letztlich sind doch alle „Corona-Helden“, die in dieser schwierigen Situation besonderes leisten, und dafür eher mehr Geld als weniger erhalten müssten! Die Frage, wer für die Schulden aufkommt, wird durch die niedrigen Zinsen erträglich gemacht. Im Übrigen steht der große ökonomisch-kulturelle Streit der letzten zwei Jahrzehnte ohnehin unentschieden: zwischen (partei- und lagerübergreifendem) amerikanischen Neo-Keynesianismus einerseits, dem von Paul Krugman bis Donald Trump öffentliche Schulden langfristig bedeutungslos erscheinen, und einem Austeritätsideal andererseits, wie es in einigen (nord-) europäischen Staaten zuletzt weithin dominierte und das diese Schulden als unfaire Belastung kommender Generationen zu vermeiden sucht.

Die zweite Frage ist ebenfalls grundsätzlich und langfristig bedeutsam, doch ist die Debatte hier offener und kontroverser verlaufen. Schon frühzeitig wurde kritisch angemerkt, dass staatliche Maßnahmen der Kompensation und Stabilisierung eine strukturkonservierende Wirkung entfalten können, die den ordnungspolitischen Grundsätzen einer Sozialen Marktwirtschaft zuwiderläuft, falsche Anreize setzt und Innovation aus der Krise verhindert. Pointiert gesagt ist das der Konflikt zwischen „freeze“ und „creative destruction“. Die gegenwärtige politische Steuerung lässt solchen Akteuren am Markt gewiss durchaus Raum, die – horribile dictu! – von der Krise profitieren, weil sie auf digitale Vertriebskanäle setzen, Software für das Homeoffice entwickeln und vertreiben oder erfolgreich ein Corona-Vakzin aus dem Labor in die Impfzentren gebracht haben. Doch auch nachdem die ursprüngliche Hoffnung auf einen „V“-förmigen Krisenverlauf längst getrogen hat, folgt die staatliche Wirtschaftspolitik ebenso wie die Erwartung der meisten Menschen der Annahme, die Wirtschaft werde über kurz oder lang wieder in die alten Spuren zurückfinden. Gutgemeinte Appelle wie die stereotyp gewordene Warnung vor „verödeten Innenstädten“ ersetzen dabei bisweilen den nüchternen Blick auf grundlegende Transformationen, die sich längst ihren Weg gebahnt haben.

Während also Joseph A. Schumpeters (1993) Modell der „kreativen Zerstörung“ in vieler Hinsicht realitätsgerechter wirkt, hat es den Nachteil, als analytische Interpretationsfigur in historischer Perspektive, also „with hindsight“, mehr zu taugen denn als politische Handlungsanleitung. Für Agrarier im späten 19. Jahrhundert oder Industrielle in den 1960er-Jahren war es subjektiv plausibel, die Gefährdung ihrer Branchen zu beklagen. Denn es schien in den damaligen Krisen unvorstellbar, dass ein wachsender Wohlstand auf der Industrie, oder später auf einem tertiären Sektor im Umfang von 70 bis 80 % der Volkswirtschaft, gegründet werden könnte. Nicht nur Stakeholder, sondern auch vermeintliche Experten haben immer wieder gegen den Strukturwandel argumentiert oder lassen sich, wie im Falle der notorischen Oxford-Studie von 2013 zum Verlust jedes zweiten Arbeitsplatzes durch die Digitalisierung (und nicht zu vergessen: der seit Karl Marx dazugehörige Dauertopos vom Verschwinden der Mittelklasse!), bereitwillig so missverstehen (Frey und Osborne 2013). Auch wenn wirtschaftliche Struktureffekte der Coronakrise schon jetzt klar absehbar sind, etwa der Ausbau der digitalen Ökonomie („Zoom-Effekt“) und der Gesundheitswirtschaft, bedeutet das nicht, dass ausdünnenden Branchen oder Geschäftsmodellen politisch der gesenkte Daumen gezeigt werden könnte. Das wäre freilich auch nicht die Aufgabe einer ordnungspolitisch richtig verstandenen Sozialen Marktwirtschaft.

## Ausblick: Staat, Wirtschaft und ökonomische Kultur nach Corona

Ein Zwischenfazit der wirtschaftlichen Situation Anfang 2021 zeigt auf den ersten Blick mehr Licht als Schatten: In einer dramatischen Herausforderung, im zeitweisen „Herunterfahren“ einer komplexen Marktgesellschaft ist es gelungen, den Arbeitsmarkt weitgehend zu stabilisieren und eine größere Insolvenzwelle – vorerst – zu vermeiden. Gegen die meisten Erwartungen liegen die Börsen im Plus; der wichtigste deutsche Aktienindex Dax hat 2020 um etwa 3,5 % zugelegt, was zugleich Vertrauen in eine weitere Erholung ausdrückt. Dass die Coronakrise, ähnlich wie schon die Banken‑, Finanz- und Währungskrise zwischen 2008 und 2011, nicht in einen wirtschaftlichen oder gesellschaftlichen Kollaps geführt hat, sondern durch ein überraschend hohes Maß an Stabilität und Kontinuität geprägt ist, hat mehrere Gründe. Erstens war die Governance der Krise, war ihre politische Steuerung wesentlich effektiver, als das in früheren historischen Krisen, etwa am Beginn des 20. Jahrhunderts, der Fall war. Demokratische Regierungssysteme und offene Gesellschaften schneiden dabei gut ab, sind andererseits aber gegenüber autoritären Ordnungen wie derjenigen der Volksrepublik China nicht per se im Vorteil. Zweitens verschafft das Wohlstandsniveau, das entwickelte Industrie- und Dienstleistungsgesellschaften am Ende des 20. Jahrhunderts erreicht hatten, offenbar einen Puffer, der Abstürze mit existentiellen Folgen wie Hunger und Armut in großen Teilen der Bevölkerung, wie in der Weltwirtschaftskrise der 1930er-Jahre, zunehmend unwahrscheinlich gemacht hat. Das sind gute Nachrichten.

Drittens aber war das hohe Maß an Stabilität und Kontinuität, wie wir sahen, ein vorrangiges Ziel der wirtschaftlichen und fiskalischen Krisenbewältigungspolitik. Die Kosten dieser Strategie jedoch sind höchstens in Umrissen erkennbar. Sie können langfristig in einem durch Konservierung verschleppten Strukturwandel ebenso hervortreten wie in den Kosten der hohen Staatsverschuldung. Zudem ändert die relative Unsichtbarkeit einer dramatischen ökonomischen Krise nichts daran, dass der Staat in der Corona-Ökonomie, um solche Abfederung, ja geradezu Krisen-Camouflage zu ermöglichen, in neuer Weise als wirtschaftlicher Akteur in Erscheinung getreten ist. Einstweilen hat sich eine neue Staats-Marktwirtschaft etabliert, von der noch unklar ist, wann und wie sie wieder rückabgewickelt werden kann. Für eine gewisse Gelassenheit spricht, dass es sich nicht um das erste Zwitterwesen zwischen Marktwirtschaft und Staatswirtschaft handelt. Die Soziale Marktwirtschaft selber entstand, woran wir deshalb zu Beginn erinnert haben, als ein solches Hybrid. Von vielen staatlichen Regulierungen hat sie sich seit den 1980er-Jahren befreit, ohne jemals den Staat über Bord zu werfen – sei es mit der Verstärkung sozialstaatlicher Arrangements, sei es durch eine spezifische deutsche Variante neoliberaler Staatlichkeit.

Historiker sind keine Zukunftsforscher, aber als Experten für Zeitverläufe haben sie eine professionelle Kompetenz für die Mischungsverhältnisse von Kontinuität und Wandel entwickelt. So lassen sich abschließend einige Hypothesen zu langfristigen wirtschaftlichen sowie wirtschafts- und sozialpolitischen Folgen der Coronakrise formulieren.

Erstens: Die unsichtbaren und bisher kaum thematisierten ökonomischen Kosten der Pandemie werden sich zeigen, auch wenn es schwer ist (und in vieler Hinsicht bleiben wird), von ihnen ein klares Bild zu gewinnen. Das gilt etwa für den öffentlichen Sektor, dessen Produktivität ohnehin kaum messbar ist. Die in den Corona-Restriktionen veränderten Leistungsprofile im öffentlichen Sektor fließen in die Schätzungen bzw. offiziellen Angaben zum Bruttoinlandsprodukt nicht ein. An manchen Stellen wird mehr gearbeitet und zugleich mehr geleistet (etwa in Gesundheitsberufen); an anderen wird mehr gearbeitet, um unter schwierigeren Bedingungen doch nur dasselbe (Produktivitäts‑) Ergebnis zu erzielen. Daneben wären aber auch Verluste durch eine krisenbedingte „hidden idleness“ zu bilanzieren, die im öffentlichen Sektor unmittelbar folgenlos bleiben kann. Gravierender sind möglicherweise die volkswirtschaftlichen Kosten durch verzögerte Bildungswege und Bildungsabschlüsse und damit den verschobenen Eintritt von Absolventinnen und Absolventen in den Arbeitsmarkt sowie in die Sozialversicherungssysteme. Ebenso wenig bildet die Entwicklung des Bruttoinlandsprodukts die krisenbedingte Umsteuerung von Ressourcen in wirtschaftliche Bereiche ab, die mehr dem „Wunden heilen“ als dem „Wohlstand schaffen“ dienen. Das gilt nahezu buchstäblich angesichts des prognostizierten Wachstums des Gesundheitssektors zugunsten der Pandemiebekämpfung und, zukünftig, einer umfassenden Vorsorge gegen neue Pandemien (IAB 2021). Aus all diesen Gründen unterschätzt der Rückgang des deutschen BIP um fünf Prozent im Jahr 2020 die realen ökonomischen Verluste durch „Corona“ vermutlich ganz erheblich.

Zweitens: Trotz eines auffälligen (und oft beklagten) Primats des Nationalstaats in der Krise setzt sich der Trend zur europäischen Vereinheitlichung fort – vielleicht verstärkt die Pandemie ihn sogar. Historische Unterschiede zwischen den nationalen Wirtschaftskulturen verblassen zunehmend; ein „deutscher Weg“ der Sozialen Marktwirtschaft oder einer spezifischen Ordnungspolitik war in der Krise kaum erkennbar. Die Europäische Union hat sich in der Krise bewährt und gehört (gegenüber ihren Mitgliedstaaten, nicht unbedingt gegenüber globalen Konkurrenten wie China!) zu den Gewinnern. Wie die Debatte über die Verteilung der Vakzine zeigt, stehen dahinter politisch-moralische Imperative der europäischen Solidarität mindestens ebenso wie „harte“ polit-ökonomische Faktoren.

Drittens: Auch wenn „Corona“ das soziale Gewebe an vielen Stellen – nicht zuletzt in den Familien, im privaten Lebensraum – bis zum Zerreißen gespannt hat, steht der Sozialstaat als Trittbrettfahrer der Krise ebenfalls auf der Gewinnerseite. Der Begriff des Trittbrettfahrers ist dabei ganz nüchtern und funktional gemeint: Nicht nur versucht der Bundesarbeitsminister, im Windschatten der Krise das Doppelprinzip des „Förderns und Forderns“ beim ALG II („Hartz IV“) zu beseitigen. Die wirtschaftliche und soziale Situation hat vielmehr Anlass zu neuen Debatten über Ziele wie eine Viertage-Arbeitswoche, das Arbeitnehmerrecht auf Homeoffice und ein garantiertes Grundeinkommen gegeben. Das alles muss nicht Wirklichkeit werden, aber kann auch nicht mehr „ungedacht“ gemacht werden. Es wird also weiterhin auf der Agenda entsprechender Stakeholder stehen und die Sozialpolitik der nächsten Jahre maßgeblich beeinflussen. Die staatliche Stützung bzw. Subsistenzsicherung der informellen Ökonomie von Künstlern, Musikern und anderen „Kreativen“ wird den Forderungen nach einem staatlichen Grundeinkommen, wie immer es am Ende heißen mag, zusätzliche Hebelkraft verleihen. De facto hat die staatliche Umsatz- und Einkommenssubstitution Elemente einer Grundsicherung schon vorweggenommen.

Viertens: Gegen die Befürchtungen, die Bewältigung der Pandemie werde die politische Bearbeitung des Klimawandels und die Verfolgung strategischer ökologischer Ziele verdrängen, ist zu vermuten, dass die Krise sich am Ende eher als Katalysator der Klima- und Umweltpolitik und im weiteren Sinne des Übergangs zu einer „ökologischen Marktwirtschaft“ erweist. Dafür sprechen freilich eher kulturelle als im engeren Sinne ökonomische Indizien. Die Pandemie hat eine mentale Zäsur gesetzt, in der es ein „Weiter so“ bzw. ein Zurück zum status quo ante kaum geben kann, etwa im Mobilitätsverhalten oder allgemein in der Bevorzugung nachhaltiger Lebensweisen. Auf einem anderen Blatt steht, dass die Konflikte darüber, was als ökologisches Wirtschaften und Investition in Nachhaltigkeit verstanden werden kann, zunehmen werden – die Tesla-Fabrik in Grünheide bei Berlin zeigt solche heraufziehenden Konflikte in exemplarischer Schärfe.

Fünftens: In einem weiten gesellschaftlichen und kulturellen Horizont könnte die Coronakrise einen Bruch mit Ordnungs- und Leistungsvorstellungen befördern, unter denen moderne Gesellschaften in den letzten Jahrzehnten operiert haben. Auch hier wäre die Krise mehr ein Katalysator als die Ursache. Denn eine Tendenz zur Verweigerung der Normen permanenter Optimierung und Mobilisierung, eine Skepsis gegenüber selbstausbeuterischen Arbeitszeitregimes in sozialen Eliten war schon früher erkennbar und verstärkt sich vermutlich besonders bei den Jüngeren. In einem ganz anderen Sinne als das häufig debattiert wird, könnte das ein Ende der Ära des „Neoliberalismus“ signalisieren: nicht als das Ende eines Marktradikalismus, den es in Deutschland nie gegeben hat, sondern als Auslaufen einer Epoche des leistungsorientierten Hyper-Individualismus. Dafür spricht auch der neue pandemische Gemeinsinn, die jedenfalls breit eingeforderte und diskursiv dominante, manchmal geradezu einen solidarischen Konformismus beschwörende Orientierung auf Zusammenhalt und Achtsamkeit (Nolte 2020). Freilich ist noch nicht klar, ob solche Marketingoffensiven der eingeforderten Solidarität und des „Füreinander“ mehr sind als der Versuch politischer Steuerung der Corona-Gesellschaft. Wer weiß, vielleicht werden darin Umrisse eines wiederum neuen Aggregatzustands der so wandlungsfähigen Sozialen Marktwirtschaft erkennbar.
